# Starch Hydrogel Films with Dual Cross-Linking: Structural and Functional Characterization

**DOI:** 10.1021/acsomega.5c10553

**Published:** 2026-02-11

**Authors:** Aline Carvalho Lopes, Ana Beatriz Klosowski, Juliana Bonametti Olivato

**Affiliations:** Pharmaceutical Sciences Department, 67883 State University of Ponta Grossa, Ponta Grossa 84030900, Paraná, Brazil

## Abstract

Hydrogels consist of three-dimensional polymeric networks with hydrophilic functional groups. This study evaluated the influence of chemical cross-linking (Cc) using citric acid (CA) and physical cross-linking (Pc) based on cooling and heating cycles as an eco-friendly alternative to conventional cross-linking techniques for starch hydrogel films. The samples were subjected to both methods to assess the potential synergistic effects of a dual cross-linking approach. Cc hydrogel films were produced using 0, 0.5, 1.5, 2.5% CA concentrations and subjected to two Pc cycles. Structural, mechanical, thermal, and morphological analyses were performed while also assessing hydration and barrier properties. Results indicated that Cc promoted the formation of cross-links and significantly stabilized the matrix. At a 0.5% CA concentration, chemical cross-linking was more effective than cooling and heating cycles, reducing solubility by up to 11% when compared to Pc sample. Kinetic swelling studies conducted for 24 h showed that CA at 2.5% achieved a stable equilibrium with a significant decrease in water uptake compared to the control, also showing a denser and more rigid network. The 1.5% CA decreased the water vapor transmission rate by approximately 190% compared to the control group in both treatments (Cc and Pc). These findings highlight that the combination cross-linking has the potential to customize the physicochemical properties of starch/xanthan hydrogels for biomedical and controlled-release applications.

## Graphical abstract

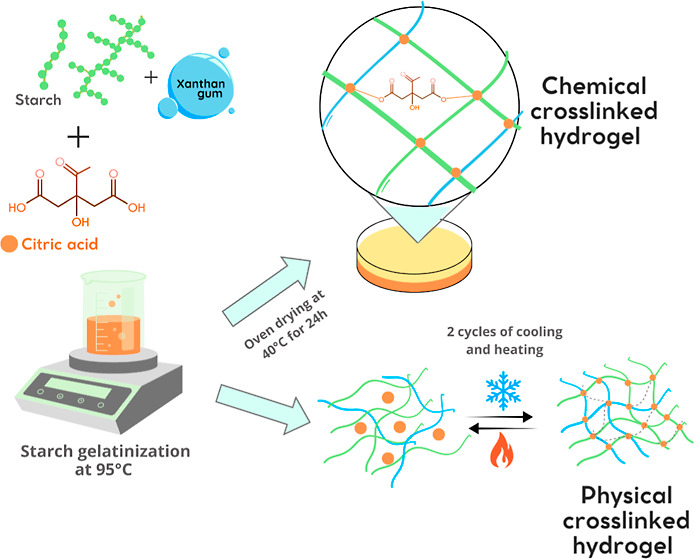

## 1 Introduction

Concerns about environmental impact and the intensive and limited nonrenewable resources have led to a renewed interest in biopolymer-based materials.^1,2^ Hydrogels are three-dimensional cross-linked networks with high swelling capacity due to their hydrophilic groups, such as –NH_2_, –COOH, –OH, –CONH_2_, –CONH, and –SO_3_H, while retaining their structural integrity without dissolving.^3,4^ Hydrogels can be used in various applications, including absorption of body fluids, acceleration of skin tissue healing, and release control of active agents during treatment.^5–7^ By incorporating two biopolymers into a hydrogel formulation, material stability is generally increased, which positively impacts its functionality.

Starch is a natural, renewable, available, nontoxic, and biodegradable polysaccharide that is widely utilized in the synthesis of biodegradable polymers, such as hydrogels.^8–11^ Starch is composed of two polysaccharides, the mainly linear amylose and the highly branched amylopectin, both made of polymeric chains containing *d* -glucopyranose residues linked by (α-1,4) glycosidic bonds and branches through (α-1,6) glycosidic bonds.^12–14^ In their native form, starch-based materials exhibit limitations, such as reduced barrier properties and high-water solubility, due to their hydrophilic character, and poor mechanical performance, resulting in brittle materials.^15–17^

Xanthan gum (XG) is a polysaccharide produced by the fermentation of *Xanthomonas campestris* bacteria.^18^ Its chemical structure consists of a linear β-1,4- *d* -glucopyranose linked, at the position 3 of the carbon in each alternating glucose residue, with a side chain composed of an acetylated *d* -mannose, *d* -glucuronic acid, and terminal pyruvated mannose.^19,20^ XG has gained significant interest in the biopolymer matrices due to its biocompatibility, processability, and nontoxic properties.^21,22^

Considering a starch/XG hydrogel, due to their distinct structures, polymers can have synergistic effects through potential conformational adjustments and chemical interactions between the polymeric chains. The introduction of a natural cross-linking agent can further improve the mechanical properties of starch/XG hydrogels. Simões et al.^22^ demonstrated that CA established ester bonds between starch and XG, resulting in an increase of 119% in elongation at break of the materials with the addition of 2.25% CA. Considering this, CA can be used as a cross-linking agent to significantly enhance the mechanical resistance of polysaccharide-based polymers, while maintaining the natural composition of the material, without the use of synthetic or toxic regular cross-link agents.^10,11,23,24^

Physical cross-linking through the freeze-thawing method has been widely used in the production of hydrogels, which promotes the formation of crystalline zones and physical interactions that occur in successive cycles of freezing at subzero temperatures and thawing at room or elevated temperatures.^25–28^ To achieve effective network stabilization, this conventional process often requires multiple cycles over several days.

As an alternative, Borges^29^ proposed an adaptation to replace the traditional freeze-thawing cycles with controlled cooling and heating cycles performed in a shorter period. This modification conserves the physical cross-linking mechanism while reducing processing time.^30^

Unlike conventional studies focusing on single cross-linking methods, this dual-approach strategy demonstrates a unique synergy that allows for precise modulation of the physicochemical properties, providing a superior balance between structural integrity and hydration capacity for starch/xanthan hydrogels. Considering this, innovative methods that combine low-cost and nontoxic techniques to produce biodegradable hydrogels for biomedical applications remain a challenge. This work aimed to compare physical cross-linking (Pc) by cooling and heating cycles with CA to promote hydrogel chemical cross-linking (Cc) as an alternative to conventional synthetic chemical agents. The effect of chemical cross-linking using citric acid with an additional physical cross-linking technique on starch/xanthan hydrogels, for potential application as biodegradable wound dressings, was evaluated with a focus on improving mechanical strength and swelling capacity.

## 2 Materials and Methods

### 2.1 Materials

Native cassava starch (moisture of 13.5% (w/w)) was obtained from Agrícola Horizonte Group (Paraná, Brazil), glycerol, used as a plasticizer, was purchased from Reagen (Paraná, Brazil). Xanthan gum (G1253, Analytical grade, batch n. 0000378768) was purchased from Sigma-Aldrich, (São Paulo, Brazil), The polymer exhibited a molecular weight of approximately 20 × 10^6^ Da, as specified by the manufacturer. Citric acid was provided by Biotec Reagents Analíticos (Pinhais, PR, Brazil).

### 2.2 Hydrogel Films Obtention

The starch/xanthan hydrogels were prepared according to the formulations presented in Table 1. The levels of CA were based on Garcia et al.^23^ and Simões et al.^22^

**Table 1:** – Formulations of the Starch/Xanthan Hydrogel Films^a^

formulations	concentrations (g 100 g^–1^)			
	cassava starch	glycerol^a^	xanthan gum^a^	citric acid (CA)
AX0	75.0	15.0	15.0	0.0
AX0.5	75.0	15.0	15.0	0.5
AX1.5	75.0	15.0	15.0	1.5
AX2.5	75.0	15.0	15.0	2.5

a In relation to the mass of starch.

The starch/xanthan hydrogels were prepared following the formulations in Table 1. For the chemical cross-linking (Cc) treatment, citric acid (CA) was used as the cross-linking agent at concentrations of 0.5, 1.0, and 2.0% (w/w, relative to the total polysaccharide weight). Xanthan gum was previously hydrated in distilled water for 12 h. This phase was then mixed with the starch/glycerol/CA aqueous solution. The mixture was heated under continuous stirring until reaching 95 °C and maintained at this temperature for approximately 5 min to ensure starch gelatinization and the onset of the cross-linking process. For each sample, 40 g of the solution was cast into circular acrylic supports (150 × 15 mm) and dried in a forced-air oven at 40 °C for 24 h.

For the physical cross-linking (Pc) treatment, the gel previously gelatinized at 95 °C (containing the same CA concentrations) was subjected to two cooling–heating cycles. In each cycle, the gel was cooled to 10 °C in a freezer and subsequently reheated to 40 °C in a thermostatic water bath. After the cycles, the samples (40 g) were cast and dried at 40 °C for 24 h, following the same procedure as the Cc treatment.

### 2.3 Thickness and Density of Hydrogel Films

The thickness of the hydrogel films was measured with a digital micrometer with a resolution of 0.001 mm and presented as the mean of ten random points for each hydrogel formulation. For the determination of density, the samples (20 × 20 mm) were stored at 25 °C in a desiccator with anhydrous calcium chloride (CaCl_2;_ ∼0% relative humidity (RH)) for 7 days and then weighed to determine their weight of the samples. To perform the density calculation, the thickness, width, and length were assessed.

### 2.4 Solubility

This test was conducted according to Lipatova & Yusova,^31^ with some modifications. The samples were previously dried for 7 days in a desiccator containing CaCl_2,_ ∼0% relative humidity (RH). After weighing, the hydrogel films were immersed in distilled water at a ratio of 30:1 (water: sample) for 48 h at 25 °C. The samples were then removed and dried in an oven at 105 °C for 3 h. The solubility of the hydrogel films was determined by calculating the weight of the samples after treatment and presented as a percentage. The test was conducted in triplicate.

### 2.5 Water Vapor Permeability (WVP)

The water vapor permeability (WVP) of the hydrogel films was determined according to the gravimetric method described in the American Society for Testing and Materials Standard (ASTM)^32^ with some modifications. Before the analysis, the samples were conditioned at 25 °C and 53% RH for 7 days. A circular permeation cell with a 60 mm internal diameter was used in the test. The interior of the cell was filled with CaCl_2_ (∼0% RH), and the cell was stored at 25 °C in a desiccator to maintain a 75% RH gradient across the film. The mass of the permeation cell was weighed every hour for a period of 8 h, and subsequently at 24 and 48 h. The water vapor permeability ratio (WVPR) was determined by using eq 1.

1 $$WVPR=\frac{w}{t}\times \frac{1}{A}$$

where weight loss (*w*) versus time (*t*) presents the angular coefficient of the linear regression, and *A* is the film permeation area (m^2^). Based on the data obtained from WVPR, it was possible to calculate the water vapor permeability (WVP), expressed in g m^–2^ h^–1^ Pa^–1^, of the hydrogels (eq 2)­

2 $$WVP=\frac{WVPR\times e}{Ps\left(\frac{{RH}_{1}-{RH}_{2}}{100}\right)}$$

where e is the mean sample thickness (m), *P*s is the vapor pressure of pure water at the essay temperature, and *RH*_1_ is the relative humidity of the desiccator, and *RH*_2_ is the relative humidity inside the permeation cell. The test was conducted in triplicate.

### 2.6 Swelling Rate (SR)

The swelling rate (SR) of the hydrogel films was determined based on the method proposed by Lee et al.,^33^ with modifications. Dried film samples (20 × 20 mm) were weighed (*W*d) and individually immersed in 30 mL of phosphate buffer solution (PBS) (pH 5.5) at 25 °C for 24 h, which simulates the slightly acidic environment of a wound. After that, an absorbent paper was used to remove the moisture on the surface, and the weight of the swelling hydrogels (Wu) was measured. The swelling rate (SR) was calculated using the eq 3. The experiment was performed in triplicate for each formulation.

3 $$SR\;\left(\%\right)=\frac{W_{u}-W_{d}}{W_{d}}\times 100$$

### 2.7 Field Emission Gun Scanning Electron Microscopy (FEG-SEM)

The surface of the materials was examined by using a FEG scanning electron microscope (Tescan model Mira 3). Before coating with a gold layer, the samples were stored at 25 °C in a desiccator with CaCl_2_ for 7 days. The coating was performed with a Sputter Coater (Quorum SC7620). Images were taken from the surface of the films, with a magnification of 1000×.

### 2.8 Mechanical Properties

A Universal Mechanical Testing machine (Shimadzu, Kyoto, Japan) was used to determine the mechanical properties of the hydrogel films. Tensile tests were based on ASTM^34^ by using 50 × 20 mm samples and a speed test of 50 mm min^–1^. The tensile strength (MPa), elongation at break (%), and Young’s modulus (MPa) were determined.

### 2.9 Thermal Analysis (TGA/DTG/DSC)

Thermal analysis of the hydrogel films was performed using simultaneous thermogravimetric (TGA), derivative thermogravimetric (DTG), and differential scanning calorimetry (DSC) techniques using Labsys Evo TGA/DTA/DSC equipment (Setaram Instrumentation). The analysis was performed with continuous nitrogen flow (rate of 30 mL/min), and the samples were scanned in a temperature range from 20 to 600 °C with a ramp of 10 °C/min. The TGA, dTG and DSC curves were obtained using Origin 8.0 software (OriginLab, Northampton, MA, USA).

### 2.10 X-Ray Diffraction

The hydrogels films were evaluated using an X-ray diffractometer (RIGAKU, Ultima IV, Tokyo, Japan), with a scan rate of 50° min^–1^ and a 2 θ range from 5° to 80°. The analysis was conducted using Cu-Kα radiation (λ-1,5218 Å), at an operating current of 40 mA and a voltage of 40 kV to observe peaks indicative of crystallinity.

### 2.11 Statistical Analysis

The data were analyzed using GraphPad Prism software (version 9.0.0, GraphPad Software, San Diego, CA, USA). The data were expressed as mean and standard deviation, followed by ANOVA analysis and Tukey’s test, with 5% variance (*p* < 0.05).

## 3 Results and Discussion

### 3.1 Thickness

The thickness of the hydrogel films (Table 2) varied significantly with the type of cross-linking treatment. Physically cross-linked films (Pc) were thicker than chemically cross-linked films (Cc), suggesting that thermal cycling affects the polymer matrix. Within each treatment, thickness also increased with higher CA concentrations (*p* < 0.05).

**Table 2:** Thickness, Solubility, and Water Vapor Transmission Rate (WVTR) of the Hydrogel Films Submitted to Two Different Treatments: Chemical Crosslinking (Cc) and Physical Crosslinking (Pc)^a^

parameter	sample	chemical cross-linking (Cc)	physical cross-linking (Pc)
thickness (μm)	AX	136.00 ± 13.00^a A^	210.00 ± 44.00^a B^
	AX0.5	155.00 ± 25.00^a,b A^	197.00 ± 35.00^a B^
	AX1.5	186.00 ± 40.00^b,c A^	182.00 ± 30.00^a B^
	AX2.5	180.00 ± 29.00^c A^	255.00 ± 56.00^b B^
solubility (%)	AX	20.48 ± 1.28^a A^	27.35 ± 0.43^a B^
	AX0.5	18.88 ± 2.42^a A^	30.92 ± 0.62^a B^
	AX1.5	27.64 ± 2.30^b A^	25.14 ± 2.16^a B^
	AX2.5	28.62 ± 1.88^b A^	27.81 ± 2.01^a B^
WVTR (g/m^2^ 24 h)	AX	42.00 ± 6.48^a A^	11.28 ± 2.40^a B^
	AX0.5	49.20 ± 8.16^a A^	16.32 ± 1.92^b B^
	AX1.5	14.64 ± 2.40^b A^	3.84 ± 3.36^c B^
	AX2.5	56.88 ± 5.52^a A^	6.24 ± 0.96^c B^

a The results are expressed as mean ± standard deviation (*n* = 3). Means followed by the different lowercase letters in the same column indicate significant differences between samples. Means followed by different uppercase letters in the same row indicate significant differences between treatments (ANOVA two-way, *p* < 0.05).

### 3.2 Density

The decrease in density with increasing CA concentration and physical treatment (Pc) (Figure 1) is related to the increase in film thickness, as the mass remains relatively constant. No significant differences were observed between films subjected to physical and chemical cross-linking overall. Chemically cross-linked hydrogels (Cc) showed higher network density at 0.5 and 1.5% CA due to effective cross-link, while physically cross-linked hydrogels (Pc) exhibited a reduction in density at AX1.5, due to CA interfering with physical gelation and chain interactions. Similar density behavior was reported by Rodrigues et al.,^45^ who evaluated starch-based films with different glycerol concentrations.

**Figure 1:**
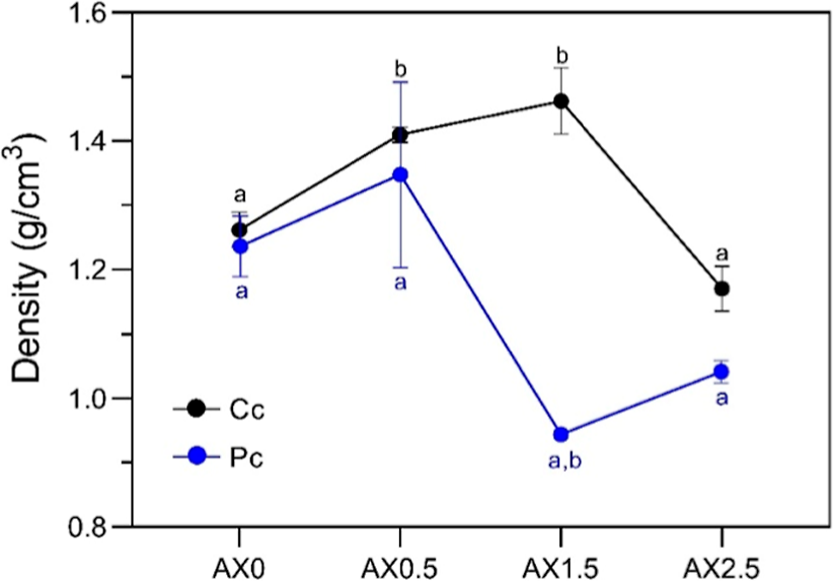
Density of starch/xanthan hydrogel films in two different treatments: chemical cross-linking (Cc) and physical cross-linking (Pc). ^a,b^ Means followed by the same letter within the same treatment are not significantly different (ANOVA one-way followed by Tukey’s test *p* > 0.05).

### 3.3 Solubility

Cross-linking agents are widely discussed in previous studies.^22,35–38^ Cross-links not only reinforce materials but also create a more compact structure. Water solubility of the hydrogel (Table 2) was significantly influenced by both cross-linking treatments (Cc and Pc) and CA concentration (*p* < 0.05) among Cc treatment.

The lower solubility observed for chemical cross-linking (Cc) compared to physical cross-linking (Pc) at lower CA concentrations (0 and 0.5%) can be attributed to the formation of robust covalent ester bonds, which provide superior network stability against water disintegration. The Pc treatment relies on physical interactions and starch retrogradation, which are more susceptible to swelling and dissolution.

Similar results were shown by Lipatova and Yusova,^31^ who investigated cross-linked starch-based films with CA and observed decreased moisture absorption.^42^ Menzel et al.,^39^ Wang et al.,^40^ and Delavari et al.^43^ demonstrated that the water solubility of starch films was significantly reduced by increasing CA concentrations. These results support that enhancing cross-linking effectively decreases water solubility in starch/xanthan materials.

### 3.4 Water Vapor Transmission Rate (WVTR)

Water vapor transmission rate (WVTR) was used to evaluate moisture control in the hydrogel films, as it directly reflects wound dressing performance under real-use conditions. Optimal dressings must adapt to varying physiological needs, providing a WVTR of 204 ± 12 g/m^2^·24 h for healthy skin, whereas injured skin demands higher rates, such as 279 ± 26 g/m^2^·24 h for first-degree burns and 5,138 ± 202 g/m^2^·24 h for granulating wounds.^44^ A WVTR between 200 and 2,500 g/m^2^·24 h provides adequate moisture without risking dehydration.^20,45^

The hydrogels showed WVTR values ranged from 3.84 to 56.88 g/m^2^·24 h (Table 2). Chemically cross-linked films (Cc) had significantly higher WVTR than physically cross-linked films (Pc), where thermal cycling promoted a more compact and organized structure, enhancing hydrogen bonding and polymer rearrangement. Increased covalent bonding in Cc films restricted polymer chain mobility and water diffusion, improving water resistance. Among all samples, AX1.5 from both Cc and Pc treatments showed the lowest WVTR (14.64 ± 2.40 and 3.84 ± 3.36 g/m^2^·24 h, respectively), suggesting a denser cross-linked matrix at this intermediate CA concentration.

Previous studies support these observations, as Lipatova and Yusova^31^ reported a slight WVTR decrease in starch films cross-linked with CA, attributed to the polymeric network formed. Ghanbarzadeh et al.^44^ showed that adding CA (0–20%, w/w) to plasticized starch films reduced water permeability due to replacement of hydrophilic OH groups with hydrophobic esters. Hydrogel films with reduced WVTR can keep a moist wound environment while absorbing exudate, which makes them suitable for wounds with low to moderate exudate.

### 3.5 Swelling Rate (SR)

To evaluate the structural stability and water absorption equilibrium of the hydrogel matrices, the swelling degree kinetics were monitored hourly (up to 6 h) and at 24 h to identify the water absorption equilibrium. The degree of swelling indicates the extent of cross-linking in a hydrogel network.^31^ According to Zou et al.,^46^ water absorption reaches equilibrium in 12 h. The results demonstrate that the time to reach equilibrium varies according to the formulation and treatment applied. In the Cc treatment, higher CA concentrations, such as 1.5 and 2.5%, promoted a rapid stabilization of the network within 4 to 6 h, as evidenced by the plateau in Figure 2-A.

**Figure 2:**
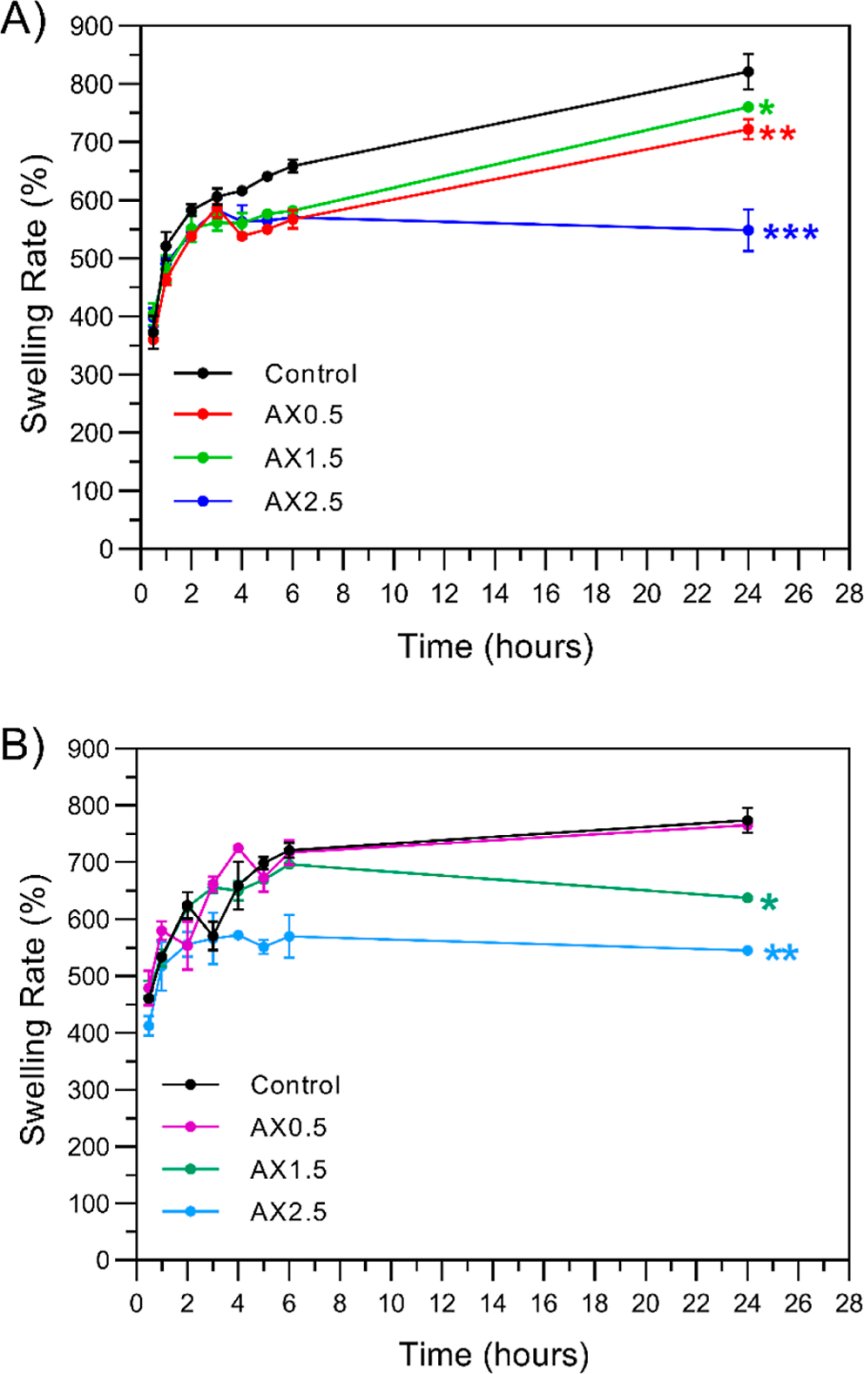
Swelling kinetics of starch/xanthan hydrogels under (A) chemical cross-linking (Cc); and (B) physical cross-linking (Pc). Markers represent the mean ± SD. Statistical significance at 24 h is indicated by asterisks (*p < 0.05; **p < 0.01; ***p < 0.001) compared to the control group (ANOVA two-way followed by Tukey’s test *p* > 0.05).

A gradual approach to equilibrium was achieved through the Pc treatment. Specifically, the 0.5% CA concentration showed no significant difference compared to the control at 24 h, suggesting that low CA levels do not substantially interfere with the physical junction zones formed by starch retrogradation. However, the 2.5% CA formulation showed a significant restriction in network expansion compared to the control (*p* < 0.0001) at the 24 h. The reduction suggests that higher concentrations of CA effectively restrict the expansion of the starch/xanthan chains, attributed to cross-link formation (Figure 3), promoting a more compact physical network. The profiles demonstrate that the cross-linking density has a significant impact on the time and capacity of the hydrogel’s steady-state hydration.^46,47^

**Figure 3:**
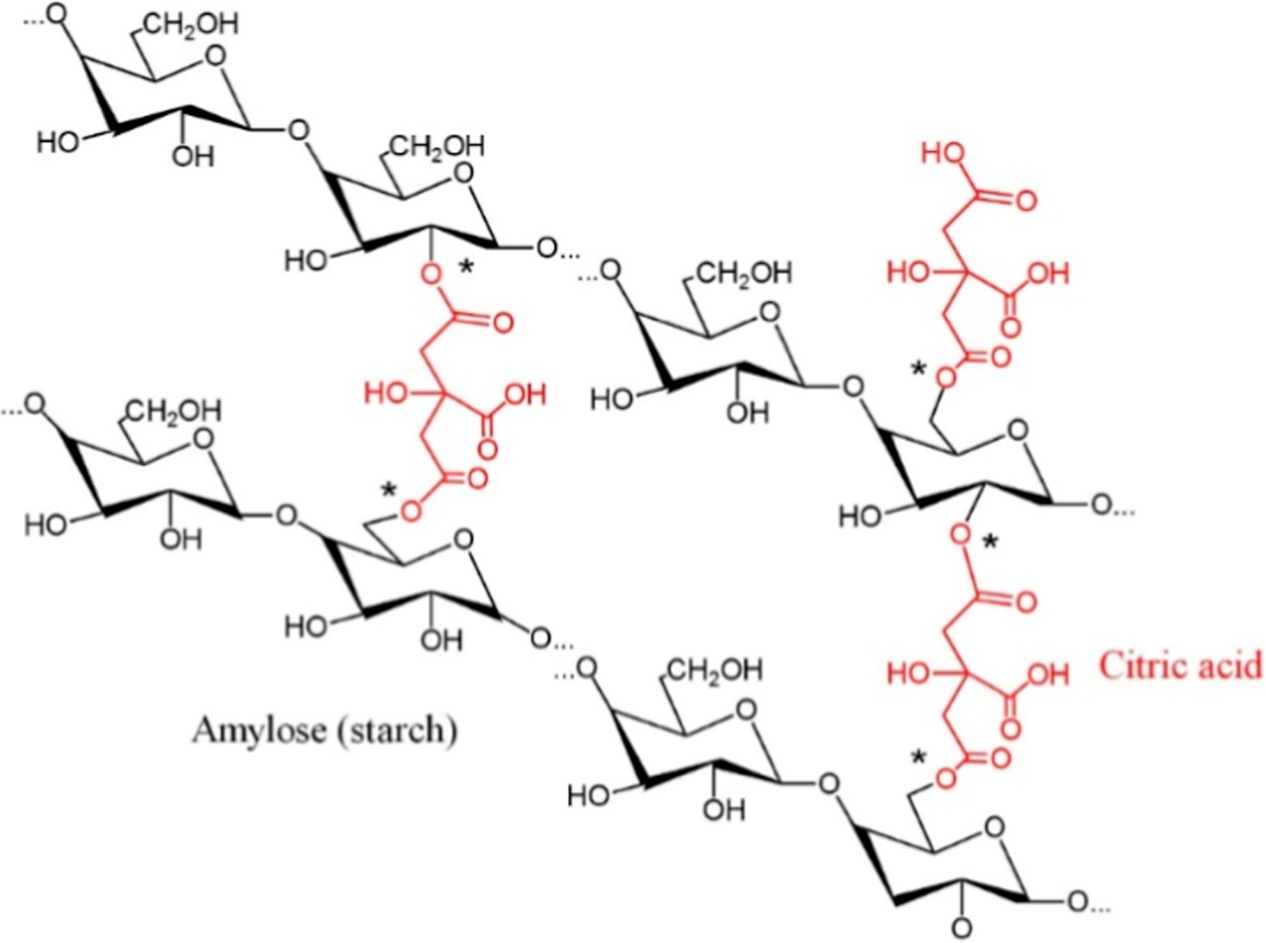
Schematic illustration of citric acid cross-linked starch. (* Shows the esterification points on starch structure).

These findings, along with solubility and density data, restrict water penetration and validate that increased CA content improves cross-linking and decreases the hydrogel’s swelling capacity, consistent with previous studies.^9,11,22,24,31,48,49^

### 3.6 Field Emission Gun Scanning Electron Microscopy (FEG-SEM)

Field emission-gun scanning electron microscopy (FEG/SEM) images of the hydrogel surfaces are shown in Figure 4. All samples exhibited a homogeneous, smooth surface without pores or visible starch granules, indicating proper gelatinization. Neither CA concentration in both cross-linking treatment (Pc or Cc) caused significant morphological differences, suggesting that these modifications do not affect microscale surface structure. Good component dispersion is evidenced by the absence of phase separation or particle agglomeration.^50^ Similar observations were reported in previous studies.^22,31,36,45^

**Figure 4:**
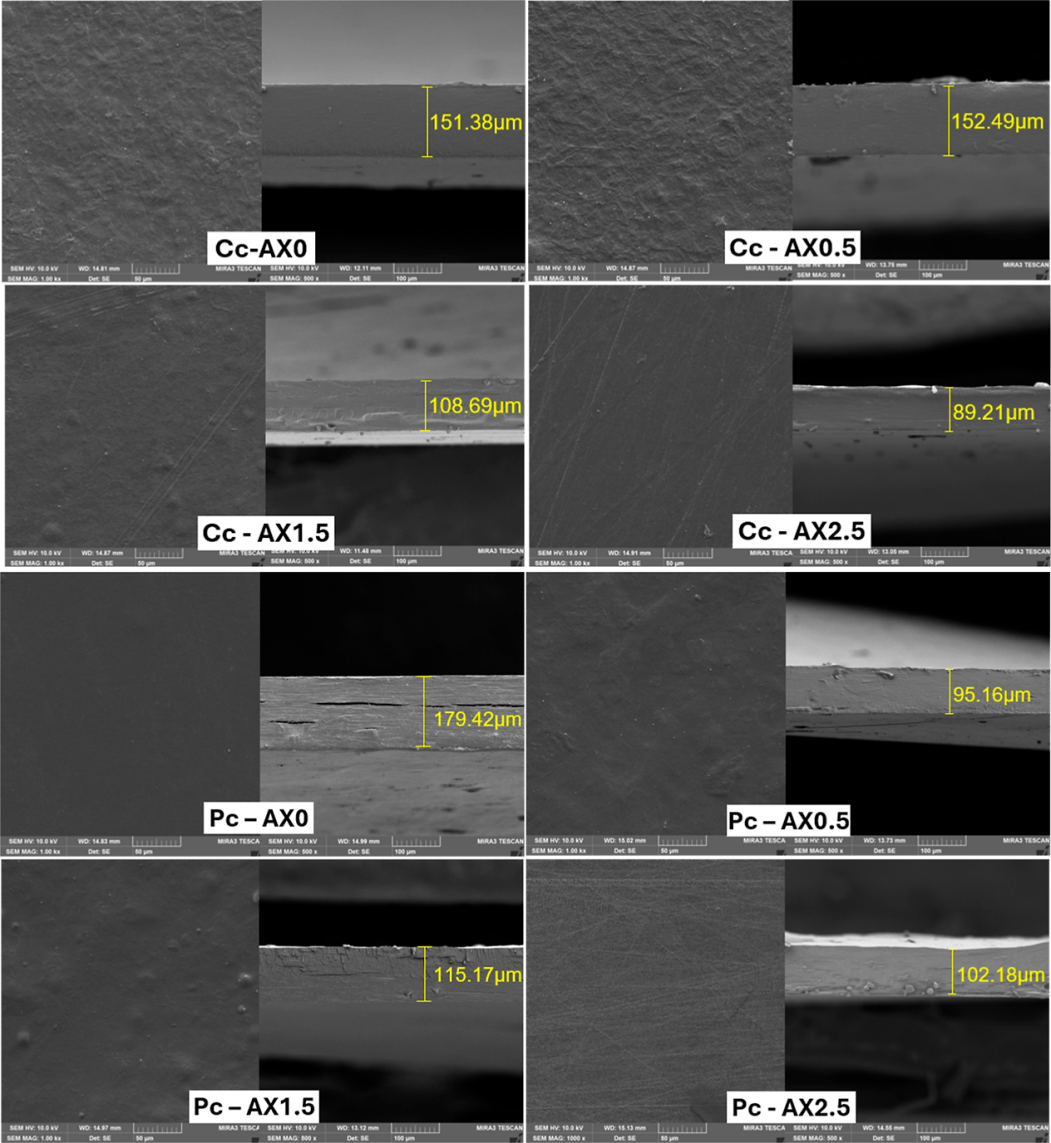
Field emission scanning electron microscopy (FEG/SEM) images of starch/xanthan hydrogel films with different CA concentrations (0, 0.5, 1.5 and 2.5%) in two different treatments: chemical cross-linking (Cc) and physical cross-linking (Pc).

### 3.7 Mechanical Properties

The percentage elongation of hydrogels is shown in Figure 5. The Cc group exhibited higher flexibility across all formulations, while samples in Pc treatment reduced elongation, suggesting that thermal cycling produces a more rigid and compact matrix, limiting polymer chain mobility.

**Figure 5:**
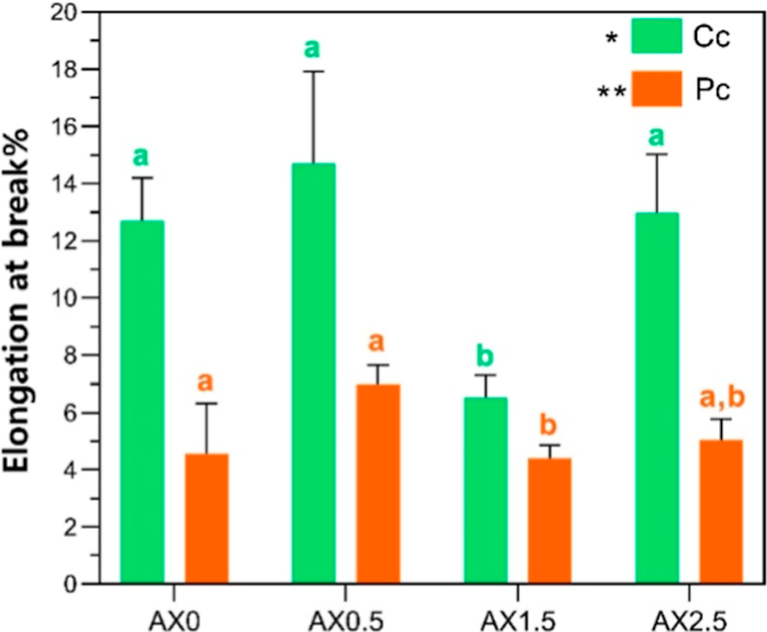
Elongation at break of starch/xanthan hydrogel films with different CA concentrations (0, 0.5, 1.5 and 2.5%) in two different treatments: chemical cross-linking (Cc) and physical cross-linking (Pc). * and ** Indicate significant differences between treatments (two-way ANOVA, *p* < 0.05). ^a,b^ Means followed by the same letter within the same treatment column are not significantly different (ANOVA one-way followed by Tukey’s test; *p* > 0.05).

At higher levels of concentration, the excess of CA acts as a plasticizer and results in a decrease in tensile strength and an increase in elongation. It is suggested that a film used for biomedical applications as wound dressings should be strong and flexible.^51^

Chemical (Cc) and physical (Pc) cross-linked samples showed no significant differences in tensile strength or Young’s modulus (Figure 6), however, variations in CA concentration within each treatment produced significant differences. In the Pc treatment, the AX0.5 sample exhibited a significantly lower Young’s modulus, suggesting that the low CA concentration (0.5%) did not provide enough cross-linking to form a rigid network.

**Figure 6:**
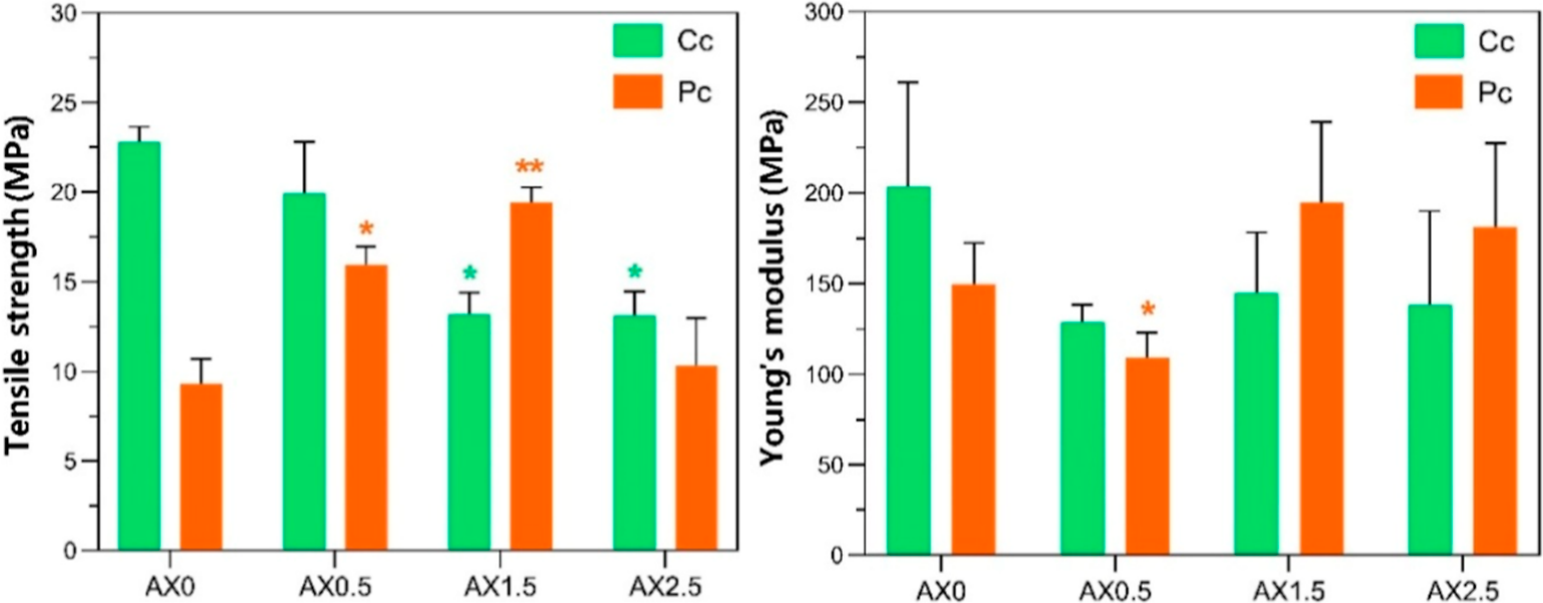
Tensile strength Young’s modulus of hydrogel with different CA concentrations (0, 0.5, 1.5 and 2.5%) in two different treatments: Chemical cross-linking (Cc) and physical cross-linking (Pc). * Statistically significant differences between samples for the same treatment (one-way ANOVA followed by Tukey’s test, *p* < 0.05).

The mechanical properties of starch-based materials can be affected by cross-linkers in different ways, depending on concentration, with higher levels reducing tensile strength and increasing flexibility.^38,40,41,52,53^ It has been reported that the incorporation of an increasing amount of CA decreases tensile strength and improves both elongation at break and Young’s modulus.^22^ The incorporation of high content cross-linkers increased the tensile strength and decreased the elongation at break of biopolymer films.^53^

### 3.8 Thermal Properties

The thermal behavior of the starch/xanthan hydrogel films was evaluated by thermogravimetric and derivative thermogravimetric analysis as shown in Figure 7 (TGA and DTG) and differential scanning calorimetry as shown in Figure 8 (DSC). Regarding TGA and DTG analyses, all samples exhibited a typical three-step degradation profile.

**Figure 7:**
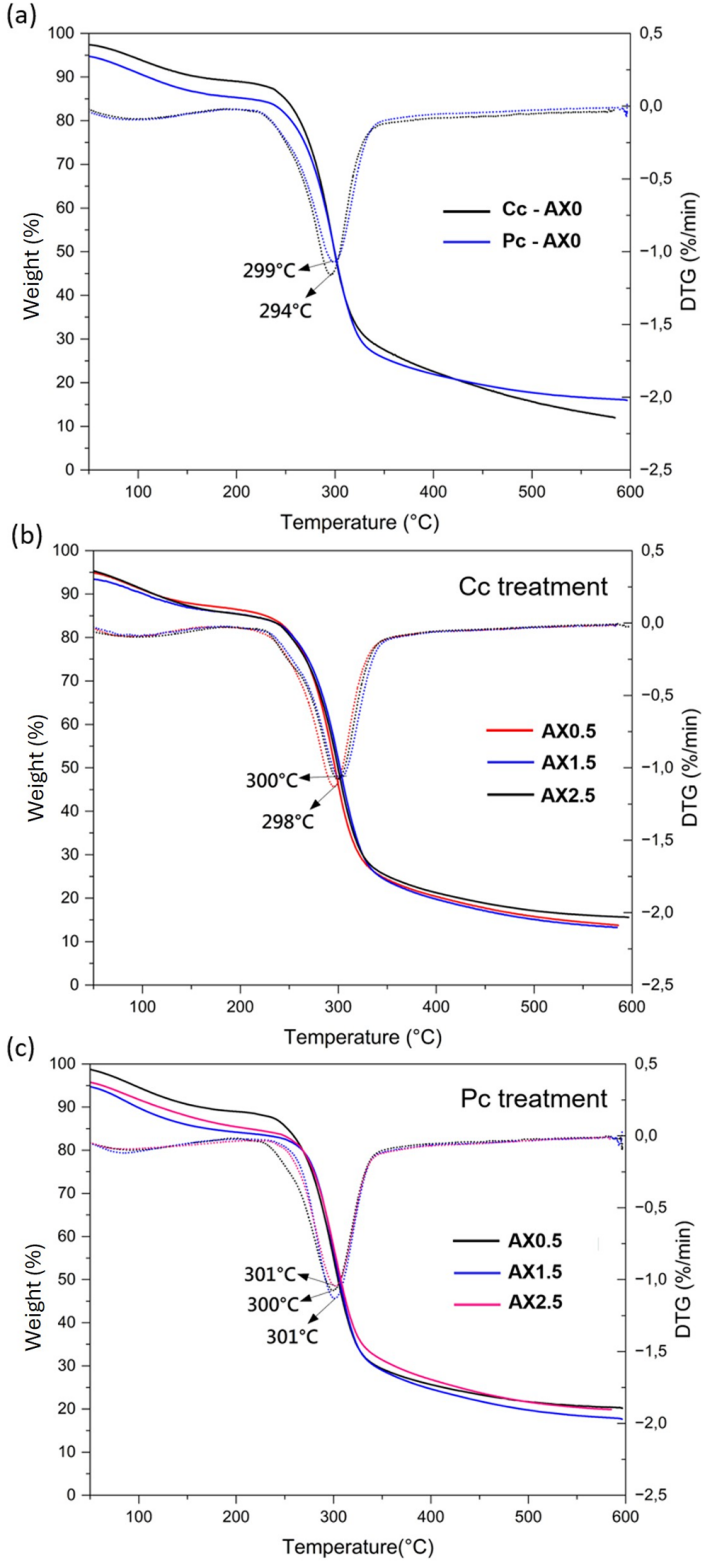
(a) TGA and DTG graphs of AX0 in two treatments (chemical cross-linking—Cc; physical cross-linking—*P*c). (b) TGA and DTG graphs of AX0.5, AX1.5 and AX2.5 from Cc treatment. (c) TGA and DTG graphs of AX0.5, AX1.5 and AX2.5 from Pc treatment.

**Figure 8:**
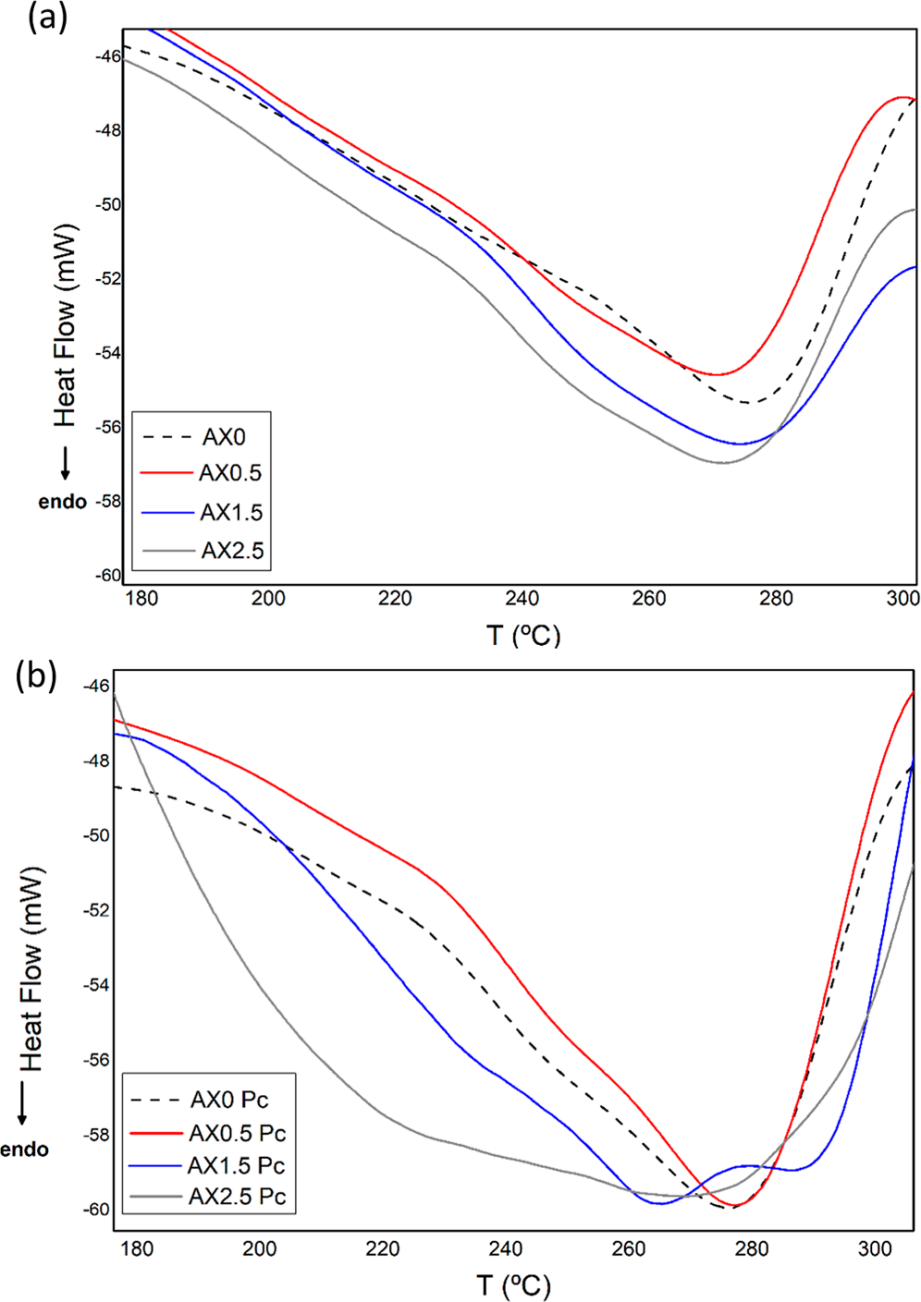
(a) DSC thermograms for hydrogels films in chemical cross-linking treatment (Cc); and (b) physical cross-linking treatment (Pc).

Between 50 and 150 °C the first weight loss is observed, which corresponds to the moisture loss. The second and most relevant weight-loss event (200–350 °C) refers to polymer degradation, involving dehydration of polysaccharide rings, cleavage of glycosidic bonds, and, in CA-containing samples, degradation of CA-derived esters. The hydrogels thermal stability, assessed by the DTG maximum degradation temperature (Tmax), showed a slight increase when CA was introduced in both treatments (Cc and Pc), with Tmax around 300–301 °C, compared to the respective controls (Cc-AX0:294 °C; Pc-AX0:299 °C), indicating modest improvement in thermal resistance. Similar CA-related decomposition peaks near 212 °C have been reported by Uranga et al.^54^ and Wu et al.^38^ The third degradation stage (>400 °C) reflects the breakdown of carbonaceous residues and the formation of char, where the degradation rate stabilizes, marking the end of major thermal processes.

The differential scanning calorimetry (DSC) curves of the hidrogels (Figure 8) were analyzed to determine the differences in the thermal behavior of the materials DSC. It is observed through the DSC curves that all materials have profiles with occurrence of endothermic peaks, with some changes in maximum temperatures and intensity, through the cross-linking technique.

In Figure 8a, the hidrogels chemically treated with CA exhibited endothermic peaks in the 240–300 °C range, corresponding to the second stage observed in the TGA/DTG analysis. This stage involves dehydration of polysaccharide rings, cleavage of glycosidic bonds, and degradation of citric acid esters in samples with CA, indicating that the heat flow signals reflect the complex thermal degradation of the polymeric matrix. Regarding physical treatment via heating–cooling cycles (Figure 8b), hydrogels indicated more intense endothermic peaks, occurring at slightly lower temperatures in AX1.5 and AX2.5 samples.

The transition temperatures (*T*_onset_ and *T*_peak_) and the enthalpy of gelatinization/melting (Δ*H*) are summarized in Table 3.

**Table 3:** Thermal Transition Temperatures (*T*_onset_, *T*_peak_, *T*_endset_) and Enthalpy of Gelatinization (Δ*H*) for Starch/Xanthan Hydrogel Films under Chemical (Cc) and Physical (Pc) Crosslinking Treatments

treatment	samples	sample weight (mg)	*T*_onset_ (°C)	*T*_peak_ (°C)	*T*_endset_ (°C)	Δ*H* (J/g)
chemical cross-linking (Cc)	AX0	15.32	271.84	280.31	289.52	14.88
	AX0.5	15.80	268.45	276.73	285.1	16.12
	AX1.5	15.14	274.12	282.86	292.34	15.58
	AX2.5	15.52	273.56	281.51	290.05	15.44
physical cross-linking (Pc)	AX0	25.20	270.92	279.45	288.15	9.84
	AX0.5	24.91	272.55	281.4	291.08	10.22
	AX1.5	25.14	252.18	263.73	274.96	12.05
	AX2.5	25.10	218.6	237.58	252.14	18.31

The DSC parameters provide quantitative evidence of the structural stabilization achieved through the dual-cross-linking strategy, highlighting the superior thermal resistance of the chemically cross-linked (Cc) hydrogels. Specifically, the AX1.5 formulation exhibited the highest peak temperature (*T*_peak_ = 282.86 °C) and consistently higher enthalpies (Δ*H*) than its Pc equivalents, confirming that covalent esterification creates a more energetically stable and suggests a stronger three-dimensional network.

Lower transition temperatures were observed in the samples from *P*c treatment, particularly for 2.5% of CA, which indicates a plasticized effect. These thermal profiles align with the solubility and swelling results, where 1.5% CA represents the optimal level for cross-linking CA, enhancing the hydrogel network integrity.

### 3.9 X-Ray Diffraction—XRD

The X-ray diffraction peaks (Figure 9) observed in the range of 17–20° (2 θ) for both Cc and Pc groups are in good agreement with results reported in the literature for starch-based polymeric films. These angles are characteristic of the B-type semicrystalline structure, which typically emerges following the gelatinization and subsequent retrogradation of starch chains during film formation.^55–57^

**Figure 9:**
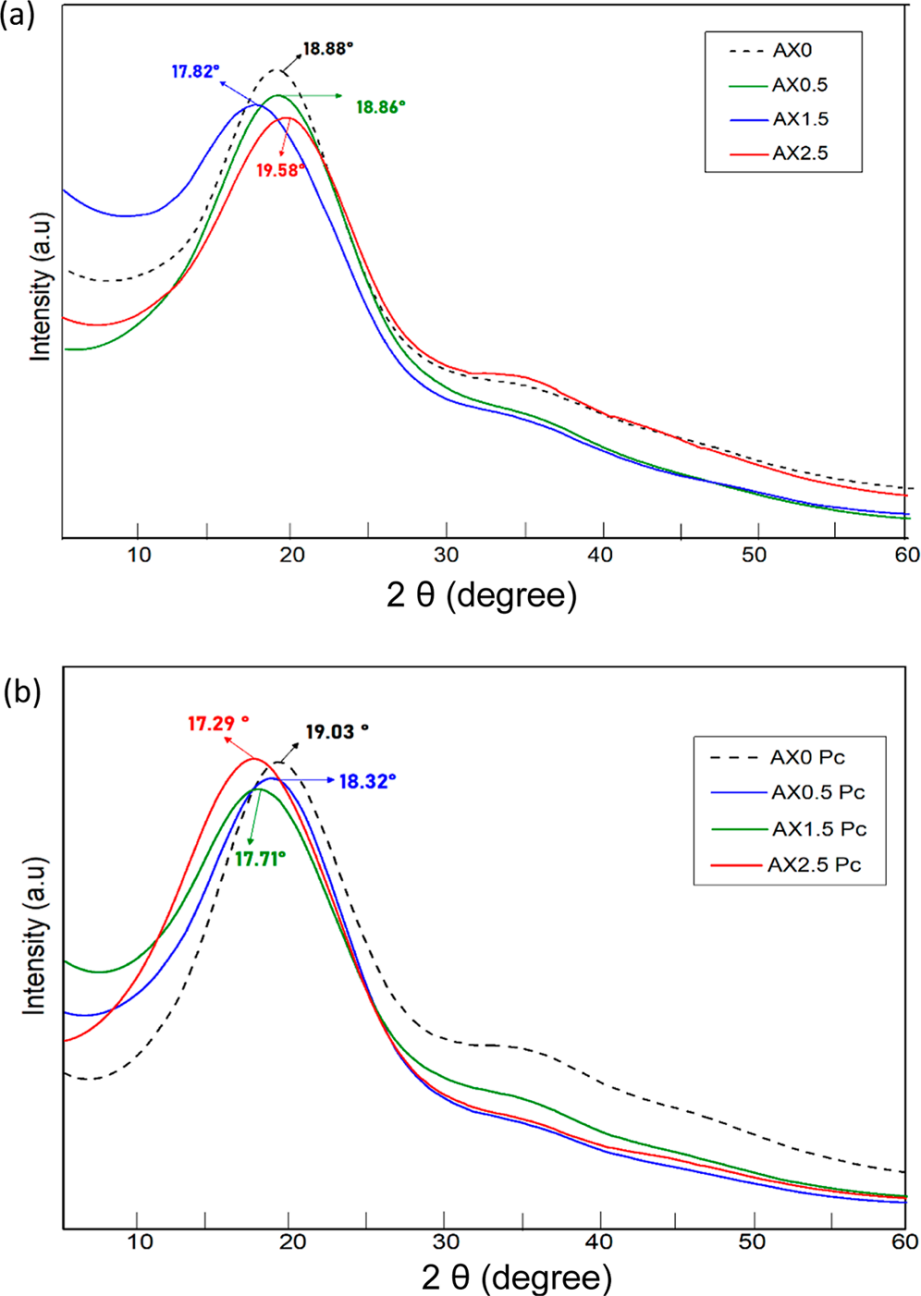
XRD patterns of starch/xanthan hydrogels: (a) chemically cross-linked samples (Cc) and (b) physically cross-linked (Pc) samples with varying CA concentrations.

Starch granules can exhibit three types of crystalline structures, A, B, and C. In the A-type crystalline structure, the packing of the double helices is more compact than in the B-type, whereas the C-type is a mixture of types A and B. Due to its density characteristics, the B-type structure can be disrupted more easily than the A-type.^58^

The Cc samples showed B-type X-ray diffraction patterns with peaks at 17–18° (Figure 9a), indicating a consistent crystalline arrangement.^55,56^ Overall, the XRD results demonstrate that both treatments maintain the essential semicrystallinity of the matrix, while the Cc treatment provides a more uniform structural organization compared to the physical cycles.^57^

## 4 Conclusion

The production of starch/xanthan hydrogel films using citric acid and thermal cycling proved effective for adjusting the properties of the materials. Variations in CA concentration and in the type of cross-linking led to measurable changes in the films’ physical and mechanical behavior, showing that both strategies can modulate the hydrogel network. Overall, chemical and physical cross-linking act as complementary approaches to control the characteristics of biodegradable starch-based hydrogels. Based on the combined trends observed in solubility, swelling, and flexibility, the formulation containing 1.5% citric acid under chemical treatment achieved the best balance between structural integrity, thermal resistance, and functional stability, suggesting its suitability for subsequent stages of material development.

These findings suggest that both strategies can be used in combination to modify biodegradable materials for several uses, with chemical cross-linking standing out as the most effective method for high-performance starch-based hydrogels.
